# Evaluating the Acceptance and Usability of an App Promoting Weight Gain Prevention and Healthy Behaviors Among Young Women With a Family History of Breast Cancer: Protocol for an Observational Study

**DOI:** 10.2196/41246

**Published:** 2022-12-16

**Authors:** Mary Pegington, Alan Davies, Julia Mueller, Rachel Cholerton, Anthony Howell, D Gareth Evans, Sacha J Howell, David P French, Michelle Harvie

**Affiliations:** 1 Division of Cancer Sciences The University of Manchester Manchester United Kingdom; 2 The Prevent Breast Cancer Research Unit The Nightingale Centre Manchester University NHS Foundation Trust Manchester United Kingdom; 3 Division of Informatics, Imaging and Data Sciences The University of Manchester Manchester United Kingdom; 4 Medical Research Council Epidemiology Unit University of Cambridge Cambridge United Kingdom; 5 Manchester Centre for Health Psychology School of Health Sciences University of Manchester Manchester United Kingdom; 6 Manchester Breast Centre University of Manchester Manchester United Kingdom; 7 Department of Medical Oncology The Christie NHS Foundation Trust Manchester United Kingdom; 8 Division of Evolution, Infection and Genomics University of Manchester Manchester United Kingdom

**Keywords:** breast cancer, weight, BMI, weight gain, health behavior, weight maintenance, women, app, ehealth, interview, mobile app, women's health, mHealth

## Abstract

**Background:**

Breast cancer is the most common form of cancer in women, and around 20% of cases are associated with factors such as adult weight gain, overweight and obesity, and potentially modifiable health behaviors including high alcohol intake, smoking, lack of physical activity, and breastfeeding. Significant weight gain occurs between the ages of 18 and 35 years; hence, this age group could benefit from weight gain prevention interventions. Population studies have reported that women at increased risk of breast cancer account for a disproportionate amount of cases. Thus, there is a particular need to target weight gain prevention and other health behavior interventions for women at increased risk. A literature review identified no evidence-based apps that cover all relevant health behaviors. With patient and participant involvement from the target population, we have developed a new app to promote healthy behaviors among young women at increased risk of breast cancer. Alongside the app, a Facebook group provides peer support, and a virtual welcome event provides an overview of the project and the opportunity to meet the research team and other study participants. The aim of the intervention is to prevent weight gain via changes to eating habits and physical activity levels, and improve other health behaviors associated with breast cancer. The app includes goal setting and self-monitoring of health behaviors and provides education about breast cancer.

**Objective:**

This study aims to assess the acceptability and usability of the app in young women at increased risk of breast cancer, and the feasibility of the study procedures for a future, larger efficacy study.

**Methods:**

Young women (n=35, age 18-35 years) at increased risk of breast cancer (>17% lifetime risk) will be recruited via 2 recruitment procedures: mailed invite from the local breast cancer family history, risk and prevention clinic, and advertisements on social media and websites. Participants will have access to the app and the private Facebook group for 2 months. They will complete questionnaires regarding their health behaviors and breast cancer risk belief at the start and end of the study, complete app rating scales in the middle and at the end of the study, and be invited to give feedback on the app during the study period. Approximately 20 participants will have a semistructured interview at the end of the study regarding their views on the app and trial procedures.

**Results:**

The trial is ongoing, and the publication of results is anticipated in 2023.

**Conclusions:**

The trial will provide evidence regarding the acceptability and usability of the newly developed app for young women at increased risk of breast cancer. Feedback obtained will be used to improve the app. The trial will also assess the feasibility of the study procedures and how these can be refined for a future efficacy study.

**Trial Registration:**

ClinicalTrials.gov NCT05460650; https://clinicaltrials.gov/ct2/show/NCT05460650

**International Registered Report Identifier (IRRID):**

PRR1-10.2196/41246

## Introduction

### The Importance of Health Behaviors for Breast Cancer Prevention

Breast cancer is the most frequent female malignancy worldwide, with over 2 million diagnoses annually worldwide and over 55,000 diagnoses in the United Kingdom [1]. These figures are predicted to increase [2] in part due to an aging population and increasing trends in modifiable breast cancer risk factors.

Recent estimates show that weight gain, excess weight, and potentially modifiable health behaviors are causally linked with a high proportion of breast cancer in the United Kingdom (~20%) [3]. The estimated attributable risks are 8% for weight gain through adulthood and overweight and obesity, 8% for high alcohol intake, and 5% for the absence of breastfeeding [3]. Other health behaviors that increase risk include smoking and lack of physical activity [4].

A recent UK study reported that a significant proportion of breast cancer cases, around 38%, occur in the 18% of women who are at increased risk (>17% lifetime risk) on the basis of their family history, +/ mammographic density, +/ hormonal factors, +/ high-risk single nucleotide polymorphisms (SNPs) [5]. Excess weight and unhealthy behaviors (high alcohol intake, smoking, unhealthy diet, and lack of physical activity) have an equal or greater effect on the relative risk for breast cancer among women with a family history compared to women without a family history of breast cancer [6-10]. Targeting health behavior interventions to women at increased risk of breast cancer is likely to have a significant impact on reducing rates because the same relative risk reduction will lead to greater absolute risk reductions.

### Current Health Behaviors Among Women at Increased Risk

Many women known to be at increased risk attend Family History, Risk and Prevention Clinics (FHRPCs) of which there are around 90 in the United Kingdom. UK guidance from the National Institute of Health and Care Excellence (NICE) recommends FHRPCs to provide advice on health behaviors to lower breast cancer risk [11]. However, our 2016 survey of 21 FHRPCs found that few do in practice, most likely due to limitations on time, skills, and resources (personal communication). Analyses of BMI and health behavior data from women at increased risk (n=136, mean age 41.2, SD 3.5 years) in our clinic at The Nightingale Centre, South Manchester, highlighted the prevalence of overweight and obesity, and unhealthy behaviors that were comparable to women in the general population [12]. Almost 60% had overweight or obesity, 30% did not meet physical activity recommendations, and 45% exceeded alcohol recommendations. Thus, there is an unmet need to provide cancer prevention health behavior programs for women at increased risk.

Our recent overview highlighted that the majority of weight gain in women occurs between the ages of 18 and 35 years [13]. Once the weight is gained, it is very difficult to lose. Hence, this project is focused on preventing weight gain and improving health behaviors among women aged 18 to 35 years.

### The Need for a Health Behavior App

Our previous interview study reported that young women (aged 25-35 years) at increased risk of breast cancer are interested in joining a program to prevent weight gain and promote healthy behaviors which could be accessed remotely, potentially via an app [14]. This was in line with the views expressed by our public and patient involvement (PPI) group of women younger than 40 years at increased risk of breast cancer who had been a healthy weight at age 18 years but had since gained at least a stone in weight (unpublished data). A program delivered via an app could also be scalable to all UK FHRPCs without putting additional pressure on each clinic.

While there are a number of weight loss apps already on the market, there are currently no apps designed to prevent weight gain. A search of the literature revealed no publications on the development of such apps; therefore, there is nothing that is currently suitable for testing in FHRPCs. Additionally, there are many breast cancer information and prevention apps, which only provide static information, for example, on risk factors and health behavior advice. They are neither interactive nor grounded in recognized psychological theory; therefore, they are unlikely to elicit behavior change [15].

### Development of the App

We have developed an app promoting weight gain prevention and healthy behaviors among young women at increased risk of breast cancer using a codesign process involving young women from the FHRPC, Manchester University NHS Foundation Trust (MFT). The development was based on the person-based approach including performing qualitative research with users in the planning stages and the creation of guiding principles which are the features of the intervention identified as central to achieving the objectives [16,17]. We held 4 web-based PPI groups between September 2020 and October 2021 with between 2 and 7 PPI participants using an iterative approach to app development. Following each meeting, the participants’ opinions were fed back to the app development team. Our multidisciplinary team includes researchers with experience of developing health apps, behavioral psychologists, dietitians, and breast oncologists. The app includes behavior change techniques found to be effective within health behavior apps in the literature, including self-monitoring [18-21] and goal setting [18,22-25], and provides education about health and breast cancer topics via an embedded microsite. Users can customize the app by choosing imperial or metric units for height and weight entries, opt to have their BMI or not have their BMI calculated after entering a weight, and choose their desired frequency for completing the 5 health logs (weight, alcohol intake, healthy eating score, smoking, and physical activity). Only weight must be completed at least monthly. Logs that are not relevant, for example, the smoking log for a nonsmoker, will not be displayed. After submitting a log, the participants are prompted to document their next target. Participants receive push notifications when their logs are due and are able to view graphs of their progress. The microsite is embedded within the app and contains information about topics such as alcohol and breast cancer, importance of fruit and vegetables in the diet, how to set health behavior goals, and how to limit weight gain in pregnancy.

### The Health Behavior Intervention: Microsoft Teams Welcome Event, App With Embedded Microsite, and Private Facebook Group

**Figure 1:**
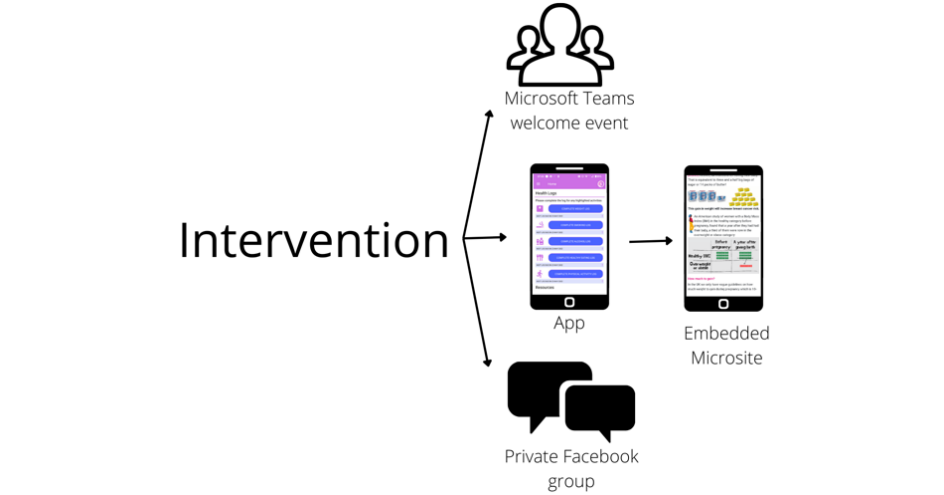
Components of the app.

The aim of the health behavior intervention is to prevent weight gain (via encouraging healthy behaviors such as healthy eating and physical activity) and improve other health behaviors associated with breast cancer, that is, reduced alcohol, smoking cessation, and breastfeeding. All of the components of the intervention are shown in Figure 1.

Alongside the app, a private, hidden Facebook group allows participants to access social/peer support within the study population for behavior change and contact with the research team. Membership of the private Facebook group is by invitation only by the research team who will also act as moderators. Only the invited members can see the list of members in the group and what members post, comment on and share within the group, and only current, invited or former members of the group can see the group’s name and description, and can find the group in a web-based search. The participants’ membership of the group and activity within the group are not visible on their personal Facebook profiles. Participants are encouraged to post within the group, and weekly posts from the research team will introduce a new weekly educational topic to promote interaction, for example, a poll or request for a recipe exchange. The research team will check the group at least once daily and reply to comments and private messages.

After consent, participants will be invited to attend one of several 45-minute welcome events hosted on Microsoft Teams with up to 10 other participants. These will provide a simple overview of the evidence for the association between weight, health behavior risk factors, and breast cancer, meet and build relationships with other study participants who will be present in the private Facebook group, and build rapport with the research team.

### Testing the Complex Intervention

Evidence generation for the developed app will follow “Evidence Standards Framework for Digital Health Technologies” guidance by NICE to Tier C [26]. App design, development, and testing have involved experienced health professionals to ensure content is accurate and up-to-date. This study will involve users for acceptability testing. The intervention is “complex” as it has a number of interacting components (information provision, social/peer support, self-monitoring, and goal setting) and a number of outcomes (weight, alcohol, physical activity, healthy eating, and smoking) [27]. Therefore, we will follow the Medical Research Council framework for developing and evaluating complex interventions, which indicates the need to assess the acceptability and feasibility of a new complex intervention before any evaluation of efficacy or effectiveness [27]. This study is focused on the evaluation of acceptability and usability and will highlight areas to be refined before the next stage of testing. This study will also give us an indication of the feasibility of some of the study procedures we will use in future studies, for example, recruiting via social media and using web-based questionnaires. Following this initial study, we will run a full feasibility study in line with the Medical Research Council framework to assess the feasibility of running a randomized, multicenter efficacy study. Adhering to these frameworks will help to ensure that the correct evidence is gathered to enable the implementation of the intervention in the NHS.

### Study Aim

The overall study aim is to assess the acceptability and the usability of the intervention for young women at increased risk of breast cancer, and the feasibility of the study procedures for a planned future efficacy study.

### Study Objectives

This study has the following 7 objectives:

1. Explore the views of users on their experience of the 2 different recruitment procedures (targeted mailshot, or social media, newsletters, and websites) and the web-based consent procedure
2. Assess recruitment data to explore how the 2 different recruitment procedures could be improved for the next study
3. Explore views of users on their experiences during and after using the app
4. Assess user data from the app including frequency and patterns of use of the different functions
5. Assemble a list of suggested changes to the recruitment and consent procedures, and to the app itself, to be considered before the next study
6. Quantify health care professional time required for administering the private Facebook chat group, and through email or private message support
7. Quantify researcher time required for the cleaning and analysis of app data

## Methods

### Overview

This is a single-arm observational study (with embedded qualitative elements). The study team includes a PPI participant who gave feedback on the protocol and all study documents that the participants will receive such as the participant information sheet and questionnaires. A total of 35 young women at increased risk of breast cancer will have access to the app and Facebook group for 2 months followed by an interview.

### Participants

The study will recruit 35 participants using the following inclusion criteria: female, age 18-35 years, living in the United Kingdom, moderate or high risk of breast cancer (>17% lifetime risk) [11], ability to communicate (written and spoken) in English, ability to download and use an app (available on both iOS and Android). In addition, the following exclusion criteria will be used: previous breast cancer (other cancers will not be excluded); previous bilateral preventative mastectomy, currently trying to gain weight; previous weight loss surgery; currently taking weight loss medication; prescribed (eg, orlistat, liraglutide, naltrexone/bupropion [Mysimba]) or other medical conditions that influence diet and weight, for example, diabetes, inflammatory bowel disease, or cystic fibrosis; current diagnosis of a psychiatric disorder, for example, bipolar psychotic disorder or current self-harm (self-report); current alcohol or drug dependency (self-report); or current or previous diagnosis of an eating disorder.

### Ethical Considerations

The study has been granted ethical approval by Wales Research Ethics Committee 3 Cardiff (reference 22/WA/0164). All participants will be asked to provide informed consent. The study will be performed in accordance with the Declaration of Helsinki. The study is registered online at ClinicalTrials.gov (reference NCT05460650). Data will be anonymized once data collection is complete but before analysis. There are no financial incentives for participating in the study.

### Study Procedures

**Figure 2:**
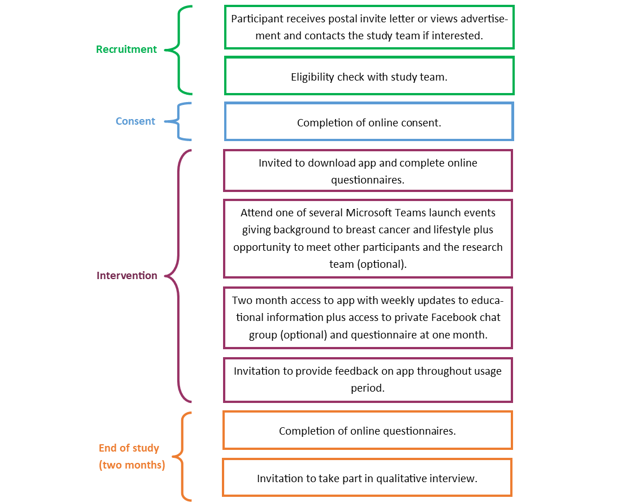
Study flow chart.

The study flowchart is detailed in Figure 2.

#### Recruitment

Participants will be recruited in 2 different ways: (1) receiving a targeted invite letter from the MFT FHRPC (estimated recruitment of 30 participants) and (2) viewing advertisements on websites/newsletters/social media platforms such as Facebook and Twitter (estimated recruitment of 5 participants).

1. Recruitment from within the MFT FHRPC: We have successfully recruited to health behavior research studies and PPI projects using postal invite letters to FHRPC participants with an uptake of between 9% and 23% (unpublished, in press, and [14]). Based on a 15% predicted uptake, 200 letters will be posted to recruit 30 women.
2. Recruitment from outside of the FHRPC: Other methods of recruitment will be used to expand diversity within the recruited population as the ethnicity of MFT FHRPC attendees is mainly White [28].

#### Eligibility Check and Consent

All participants will have a telephone eligibility check. Breast cancer risk for participants from MFT FHRPC does not need to be verified as this has already been calculated using the Tyrer-Cuzick method [29]. Participants recruited from outside of the FHRPC will be deemed at increased risk based on their family history of breast and ovarian cancer as per NICE guidelines for referral to secondary care [11]. These women will be advised that they could be at increased risk due to their family history and therefore may meet the criteria for referral into secondary care and will be advised to seek a referral to a local FHRPC via their general practitioner. Following the eligibility check, participants will be asked to complete a web-based informed consent form. In order to report uptake to the study and reasons for screen fail, we will ask interested women who are not eligible if we can store their answers to the eligibility screen. If women consent to this, the information will be anonymized prior to storage.

#### Intervention

**Figure 3:**
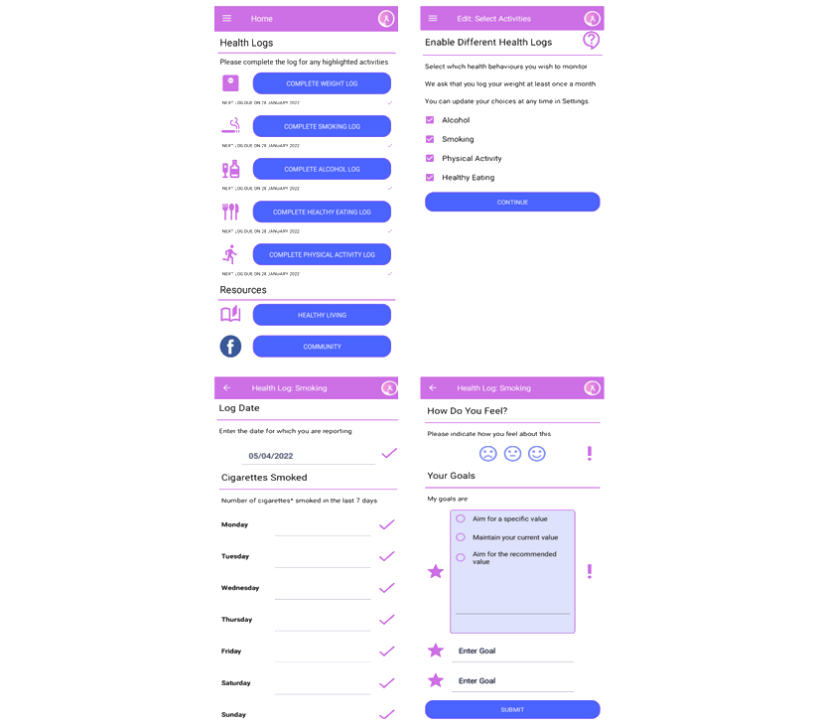
App screenprints.

Participants will be invited to attend a Microsoft Teams (Microsoft Corporation) welcome event and will have 2 months’ access to the app (Figure 3) and the private Facebook group.

#### Questionnaires

All questionnaires will be hosted electronically on Qualtrics XM (Qualtrics LLC) and completed following the timeframe in Table 1.

**Table 1:** Timeframe of study questionnaires.

Questionnaire	Baseline	1 month (midstudy)	2 months (end of study)
Demographics and Health Behaviors Questionnaire	✓	N/A^a^	N/A
Breast cancer risk beliefs questionnaire	✓	N/A	✓
Mobile Application Rating Scale [30] questionnaire	N/A	✓	✓
App feedback questionnaire	N/A	✓*Optional* as and when the participant wishes to give feedback on the app during the study	✓*Optional* as and when the participant wishes to give feedback on the app during the study

^a^N/A: not applicable.

### Outcome Measures

Outcome measures for the study and how they will be evaluated are detailed in Table 2.

**Table 2:** Outcome measures and how they will be evaluated.

Process to evaluate and method of data collection		Aims to explore	Method of analysis/data to be presented	Timepoint of analysis
**Recruitment methods**				
	Recruitment data	Recruitment source of the participants	Recruitment data, for example, percentage response to mailshot, percentage uptake, and breakdown of numbers recruited via each method	At the end of recruitment phase
	Interviews	Acceptability of recruitment procedure	Analysis of qualitative interviews	Interviews at the end of study
**Consent method**				
	Interviews	Acceptability of consent procedure	Analysis of the qualitative interviews	Interviews at the end of the study
**App: participant views**				
	Interviews, final questionnaire, app feedback questionnaires, and feedback received by email	Acceptability of the app, barriers and facilitators to engagement, likes and do not likes within the app, usability, and likelihood of extended use	Analysis of qualitative interviews, analysis of final questionnaire, and app feedback questionnaires, for example, functions most liked, suggested changes	Middle, during, and the end of the study
**App: participant usage**				
	App usage data	Frequency of use, pattern of use across the 2-month interaction with components within app	Analysis of app analytics: number of times visited the app, clicks on links within the app to external sites, clicks from notifications, clicks on help buttons, flow through the app (which screens in which sequence), duration spent on each page/on the app in total	End of study
**App: completion of information including logs**				
	Download of completed information including logs	Correct completion of the settings information and completion the frequency of the logs	Actual versus chosen frequency of completion of logs and change over time; any errors in information inputted, for example, kg entered as stones and pounds; number of logs completed for each health behavior	End of study
**Health care professional time required**				
	Health care professional time logs	Health care professional time required for moderating Facebook chat group and through email or private message support	Breakdown of health care professional time spent in total and per person on moderating Facebook chat group, responding to private messages and emails	End of study
**Engagement with the Facebook group**				
	Download of data from the group	Number of participant interactions in the Facebook group, pattern of interactions over the 2 months	Analysis of Facebook download	End of study
**Time needed to collect, clean, and analyze data**				
	Staff time logs	Estimate of staff time and costs for larger study	Breakdown of staff time spent on the study, including cleaning the app data	End of study

#### Interviews

A qualitative researcher will undertake up to 20 semistructured interviews at the end of the 2-month study. Sampling will be purposive, aiming to obtain women with a range of ages, ethnicities, and both heavy and light engagers with the app. The interviews will take place face-to-face or over Microsoft Teams and will be digitally audio-recorded and transcribed verbatim. The interview schedule (Multimedia Appendix 1) explores the participants’ experience of using the app, the usability of this app, whether it has been useful to them in changing their health behaviors, and aims to understand how the app may have influenced health behaviors or feelings toward breast cancer. It is predicted that a maximum of 20 interviews will be required, but a decision to stop further interviews will be made when no novel insights appear in the interviews according to the concept of “information power” [31].

### Statistical Analysis

This study is to assess the acceptability and usability of the app before a larger feasibility study is planned and as such is not powered according to the outcome measures of that larger study. The study will recruit 35 women, of which up to 20 will be interviewed. The study sample size of 35 will allow for a 20% dropout and selection of a range of women to interview, for example, both heavy and light engagers with the app. The study is not powered to assess the efficacy of the app at changing health behaviors and preventing weight gain, but 2 months of usage by ≥28 users will enable the quantitative data to give helpful indications of changes required to the app before a larger efficacy study.

#### Quantitative Analysis

Key baseline information from the demographics and health behaviors questionnaire will be presented, for example, smoking status, alcohol intake and physical activity in the previous week, age, living circumstances, ethnicity [32], education level, employment status, sociodemographic status (deprivation score: English Indices of Multiple Deprivation derived from full postcodes [33]), number and ages of children, and previous attendance at FHRPC using mean (SD), median (25th-75th percentile), and n (%) as appropriate.

Data from the private Facebook group will be extracted by copying and pasting it into Excel and anonymized. Data will be comments, posts (or descriptions of posts in the case of photo or video posts), and numbers of reactions. The use of an application programming interface will not be considered for this small study. Quantitative data will be presented, for example, the number of comments in reaction to weekly educational posts (total, average number per post and range, subjects evoking the highest number of comments, and number of users commenting), number of posts by participants, number of likes and reactions (total, average number per post and range, subjects evoking the highest number of reactions, and number of users reacting), and number of private messages to the moderators (number of users messaging and total number of messages received and sent) [34]. No qualitative analysis is planned on the text.

App usage data (number of times visited the app, clicks on links within the app to external sites, clicks from notifications, clicks on help buttons, flow through the app [which screens in which sequence], duration spent on each page/on the app in total) will be presented descriptively.

#### Qualitative Analysis

Transcripts will be analyzed using thematic analysis [35]. The analysis will be inductive: open-ended, exploratory, and driven by the data. Thematic analysis is free from theoretical bonds and is therefore adaptable to a wide range of methodologies. This freedom of epistemology means that the qualitative data from this study can provide a parallel and complimentary perspective to the other methods of data collection being used in this study. Thematic analysis can account for both individual and group consensus so that both convergent and divergent experiences across the corpus of the data can be taken into account as the process of analysis involves searching for all salient themes that emerge from the data. The analysis will be conducted by the qualitative health psychology researcher and codes discussed, revised, and refined in conjunction with DF.

## Results

The study is currently ongoing, recruitment is predicted to be complete by the end of January 2023, and the results are expected to be submitted for publication by the summer of 2023.

## Discussion

### Expected Findings

We have developed an intervention including a novel weight gain prevention and health behavior app with PPI participants. This study is aiming to assess the acceptability and usability of the app for young women at increased risk of breast cancer, and the feasibility of study procedures for a planned future efficacy study. Through its objectives, this study will provide evidence for changes required to the study and the intervention that should be implemented in a future study. We are following recognized frameworks in order to enable future implementation in the NHS.

### Previous Studies

The literature reveals a paucity of studies using evidence-based smartphone interventions for breast cancer risk reduction. A 2014 review by Mobasheri et al [36] highlighted the lack of evidence-based content in apps related to breast health and found potential safety concerns in 15.7% (29/185) of the apps they reviewed. In 2016, Coughlin et al [37] collated the evidence regarding modifiable behavioral risk factors that should be included in the development of apps for breast cancer risk reduction. They concluded that an app should address multiple modifiable risk factors and different apps may be required to effectively target different populations such as younger women. They highlighted the importance of including proven behavioral techniques such as goal setting. They found that no evidence-based apps currently exist and they planned to develop and test an app but as yet have not published further work. Giunti et al [38] identified 61 apps related to primary or secondary breast cancer prevention of which over 80% were developed by individuals or small-to-medium enterprises, and they, again, raised concerns about the lack of medical professional involvement in app development, many of which aim to sell products or link with paid services [38]. A 2019 systematic review of app studies that aim to reduce breast cancer risk did not find any that addressed all of the necessary behavioral risk factors and concluded that more research is required, especially for apps relevant to young women [39].

### Limitations

This 2-month study will be unable to assess app usage over a longer timeframe. The questionnaires and interviews will, however, inform whether longer-term use is likely, and changes that are required to improve engagement and longevity that will be employed in the subsequent feasibility study. The study will not attempt to establish the effectiveness of the intervention for modifying behavior. A longer study duration is required for this. Nor will the study assess changes to breast cancer risk due to behavior modification or weight change, as current risk models are unable to calculate the effect of behavioral changes such as smoking reduction/cessation or changes in physical activity levels on future breast cancer risk.

### Planned Future Steps

The findings of the study will be published. If this study shows that the app is acceptable to the target audience, it will be used in a future planned feasibility study. Any changes required to the app, Facebook page, or trial procedures that have been highlighted by this acceptability study will be completed before the feasibility study. The feasibility study will in turn inform the viability of a larger, definitive multicenter randomized controlled trial to test the effectiveness and cost-effectiveness of the app for the prevention of weight gain, and optimization of health behaviors related to breast cancer among young women at increased risk.

### Conclusions

Over 55,000 women are diagnosed with breast cancer in the United Kingdom each year, and many of these cases could be prevented through changes to health behaviors and limiting weight gain. Consideration of the existing literature surrounding health behavior interventions and breast cancer risk reduction, along with input from PPI groups, has led us to develop an app for use in young women at increased risk of breast cancer. In this initial study, we will assess whether the app is acceptable to the target population. The study will add to the literature on the development of interventions for the reduction of breast cancer risk in women at increased risk of the disease. If successful, we plan to run a larger efficacy study. By following recognized frameworks for intervention development, we will increase the likelihood of developing an intervention that can be successfully rolled out within the NHS.

## Appendix

Multimedia Appendix 1 Interview schedule.

## Abbreviations

FHRPC: Family History, Risk and Prevention Clinic
MFT: Manchester University NHS Foundation Trust
NICE: National Institute of Health and Care Excellence
NIHR: National Institute for Health Research
PPI: public and patient involvement
SNP: single nucleotide polymorphism

## Data Availability

After data analysis is complete, the fully anonymized data will be uploaded to the University of Manchester institutional research data repository and will remain there indefinitely and will be freely available.
