# Correction: Evaluating the Acceptance and Usability of an App Promoting Weight Gain Prevention and Healthy Behaviors Among Young Women With a Family History of Breast Cancer: Protocol for an Observational Study

**DOI:** 10.2196/47765

**Published:** 2023-04-04

**Authors:** Mary Pegington, Alan Davies, Julia Mueller, Rachel Cholerton, Anthony Howell, D Gareth Evans, Sacha J Howell, David P French, Michelle Harvie

**Affiliations:** 1 Division of Cancer Sciences The University of Manchester Manchester United Kingdom; 2 The Prevent Breast Cancer Research Unit The Nightingale Centre Manchester University NHS Foundation Trust Manchester United Kingdom; 3 Division of Informatics, Imaging and Data Sciences The University of Manchester Manchester United Kingdom; 4 Medical Research Council Epidemiology Unit University of Cambridge Cambridge United Kingdom; 5 Manchester Centre for Health Psychology School of Health Sciences University of Manchester Manchester United Kingdom; 6 Manchester Breast Centre University of Manchester Manchester United Kingdom; 7 Department of Medical Oncology The Christie NHS Foundation Trust Manchester United Kingdom; 8 Division of Evolution, Infection and Genomics University of Manchester Manchester United Kingdom

In “Evaluating the Acceptance and Usability of an App Promoting Weight Gain Prevention and Healthy Behaviors Among Young Women With a Family History of Breast Cancer: Protocol for an Observational Study” (JMIR Res Protoc 2022;11(12):e41246) the authors noted one error.

Reference 15 originally appeared as:

*Cacciamani GE, Aron M, Gill I, Desai M. Re: oncological outcome according to attainment of pentafecta after robot-assisted radical cystectomy in patients with bladder cancer in the multicentre KORARC database. BJU Int 2020 Nov;126(5):644-645. [doi: 10.1111/bju.15222] [Medline: 32870582]*

This has been changed to:

*Michie S, Johnston M, Francis J, Hardeman W, Eccles M. From Theory to Intervention: Mapping Theoretically Derived Behavioural Determinants to Behaviour Change Techniques. Applied Psychology 2008 July;57(4):660-680. [doi: 10.1111/j.1464-0597.2008.00341]*

The correction will appear in the online version of the paper on the JMIR Publications website on April 4, 2023 together with the publication of this correction notice. Because this was made after submission to PubMed, PubMed Central, and other full-text repositories, the corrected article has also been resubmitted to those repositories.

