# Supplementary material for: Evaluating the Acceptance and Usability of an App Promoting Weight Gain Prevention and Healthy Behaviors Among Young Women With a Family History of Breast Cancer: Protocol for an Observational Study
Source: JMIR Res Protoc. 2022 Dec 16;11(12):e41246. doi: 10.2196/41246 (PMC9804094; doi:10.2196/41246)
Supplement: Multimedia Appendix 1 [file resprot_v11i12e41246_app1.docx]

**Supplementary Information 1: Interview schedule**

| Introduction – consent and opportunity to ask questions   - Thank participant for time & introduce self - As described in the Participant Information Sheet we are asking for the views of people who have been using the [app] (clarify if needed). We expect the interviews will last no longer than an hour. - If at any time during the interview you do not wish to answer a question that’s okay. - I would like to record our conversation. The recording will be typed out, but everything you say will be anonymous. Your name and any other names or locations you mention will be taken out, so that if someone read your interview, they would not know who you are. - If, at any stage, you wish to stop or pause the audio recorder, please let me know. - Do you have any questions at this stage?   *Turn on recorder: Can I ask you to please confirm that you are happy to take part in the interview and that you are happy for me to record our conversation?*  Study recruitment/general understanding of participant health behaviours   1. How did you hear about the study? Via invite in the post or online? 2. What interested you/drew you to take part in the study?    1. Can you tell me if there were any other influences on you taking part in the study? E.g. family member, FHRPC staff, etc. 3. Did you have any questions before you consented to take part/were these adequately answered by the research team? 4. How did you find the online consent process? 5. On a scale of 0-10, how do you feel you manage your health behaviours (your weight, physical activity levels, alcohol, healthy eating) in general?    - 1. Why that number?      2. What do you feel you do well?      3. What do you feel you don’t do as well? 6. Do you think changing your own health behaviours can change your breast cancer risk?     Usability/acceptability of app   1. On a scale of 0-10, how easy did you find downloading the app and the registration process?    1. Why this number? What was easy/difficult?    2. Did you enlist any support in getting the app set up? If so, who? If not, why not?    3. Did you take part in the Microsoft Teams starter group session?       - 1. If so, why? What were your thoughts on the session?         2. If not, why not?         3. [If attended] is there anything about the session that you feel would be useful to change? 2. Usage of app over intervention - Ease of use    - 1. How easy was the app to use, when compared with other apps on your phone?      2. How easy was the process of inputting your PA/diet/other information into the app?      3. Frequency of logging information - how frequent and why?         1. Has anything got in the way of you logging at the frequency you chose to start with?         2. What do you feel comfortable logging? What don’t you feel comfortable logging? What would make you feel more comfortable logging?   d. Did you experience any issues with the app (i.e., working slowly, crashing)? If yes, what did you experience?   1. Usage of app over intervention - Usefulness/acceptability    1. What parts of the app are the most useful for you? (May need to probe and remind Ps of the areas of the app)    2. What parts of the app are least useful for you?    3. How did you react to the educational content e.g., weekly updates?    4. Did you use the Facebook support group? If so, why/how important was this to you? If not, why not? [If used - what could we do to run this group better?]    5. How would you feel about receiving feedback on how changes to your health behaviours are changing your personal risk of breast cancer and other diseases?    6. (FHRPC participants only) How would you feel about the Family History Clinic clinicians (doctors and nurses that you may have met in clinic) looking at your health log data?   How do you feel about being able to contact a health care professional from the Family History Clinic via the app?   1. Behaviour change    1. What have you learnt about breast cancer and health behaviours since you have started using the app?    2. What, if anything, has changed the way you manage your health behaviours in the last two months (or however long they’ve been using the app)?    3. How do you feel you have managed your health behaviours over the last two months, specifically?    4. Looking back over the last two months, has your perception of your breast cancer risk changed? Why/why not?    5. Can you tell me about anything that has happened in your life while you have been using the system (i.e., past two months) that may have affected either:       1. Your use of the system (e.g., if they stopped/started using app following a life event)       2. Managing your health behaviours in general (e.g., life event, social aspects) 2. Motivation to use app long-term   How would you feel about being able to compare your weight and other health log results to those of other women using the app?  Are there any features that we haven’t discussed that you’d like to see included in the app?  How would you feel about the idea of linking the app to wearables (e.g., apple watch, Fitbit)  Final comments   1. Is there anything we haven't talked about that you'd like to mention? |
| --- |
